# Longitudinal spectral domain optical coherence tomography changes in eyes with intraocular lymphoma

**DOI:** 10.1186/1869-5760-3-59

**Published:** 2013-09-08

**Authors:** Hyun Soo Jang, Yasir J Sepah, Raafay Sophie, Millena G Bittencourt, Daniel Ferraz, Mostafa Hanout, Hongting Liu, Diana V Do, Quan Dong Nguyen

**Affiliations:** 1 Retinal Imaging Research and Reading Center, Wilmer Eye Institute, Johns Hopkins University School of Medicine, Baltimore, MD, USA; 2 Stanley M. Truhlsen Eye Institute, University of Nebraska Medical Center, Omaha, NE, USA; 3 Department of Ophthalmology, University of São Paulo, São Paulo, Brazil

**Keywords:** Spectral domain optical coherence tomography (SD-OCT), Primary intraocular lymphoma, Primary retinal lymphoma, Primary vitreoretinal lymphoma

## Abstract

**Background:**

Cases of patients with primary intraocular lymphoma (PIOL) were retrospectively analyzed to describe the longitudinal intra-retinal morphological changes in PIOL as visualized on images obtained by spectral domain optical coherence tomography (SD-OCT).

**Results:**

In a retrospective case series, Heidelberg Spectralis SD-OCT images obtained in the longitudinal evaluation of patients with biopsy-proven PIOL were analyzed and assessed. The images were graded for the presence of macular edema (ME), pigment epithelial detachment (PED), subretinal fluid (SRF), and hyperreflective signals. SD-OCT scans of five eyes from five patients were assessed. Patients showed signs of inflammation, such as ME and SRF, which were resolved with treatments in some cases. Hyperreflective signals were found in all eyes in the form of nodules or bands across the retina, with the highest frequency of appearance in the ganglion cell layer, inner plexiform layer, photoreceptor layer, and retinal pigment epithelium; such signals increased with the progression of PIOL.

**Conclusion:**

SD-OCT may be employed to monitor the progression of PIOL. Hyperreflective signals on OCT may correspond with increase in disease activities, along with other findings such as ME, PED, and SRF.

## Background

Primary intraocular lymphoma (PIOL) is a subset of primary central nervous system lymphoma (PCNSL). It is a rare non-Hodgkin's lymphoma that usually involves the retina and vitreous, mainly consisting of large B-cells. PIOL represents about 1% of non-Hodgkin's lymphomas, 1% of intracranial tumors, and less than 1% of intraocular tumors [1]. It has been reported that 15% to 25% of PCNSL cases involve the eye; these may initially be present without concurrent central nervous system (CNS) involvement [2,3].

PIOL is the most common neoplastic disease that masquerades chronic posterior uveitis [4]. When misdiagnosed as uveitis, patients may initially respond to corticosteroids, which further contributes to the diagnostic challenge [2]. PIOL may also manifest in the anterior segment as keratic precipitates, cells, and flare [5]. The identification of atypical lymphoid cells in the eye is necessary to make the diagnosis [6,7].

Assessment of PIOL using ultrasonography, fluorescein angiography, indocyanine green angiography, and optical coherence tomography has been reported with a common finding of intra- and sub-RPE (retinal pigment epithelium) infiltrates [2,8]. Additionally, a few case reports of PIOL have utilized spectral domain optical coherence tomography (SD-OCT) to capture high-resolution cross sections of the retina [9-11]. However, the common patterns of SD-OCT changes in PIOL have not been established given the rarity of the disease and the relative novelty of SD-OCT imaging. To the best of our knowledge, progression of PIOL utilizing SD-OCT has been reported in one case report [11]. We herein report patterns of anatomic changes as visualized by SD-OCT in five patients of PIOL over a period of 161 ± 61 days.

## Methods

An informed written consent that was approved by the Institutional Review Board of Johns Hopkins University was obtained from all patients included in the study.

Patients with biopsy-proven PIOL who had been managed by one of the authors (QDN) at the Wilmer Eye Institute and followed using SD-OCT were included in the index retrospective case series. Patients either received systemic high-dose methotrexate (MTX), systemic rituximab (RTX), and/or intravitreal MTX and RTX.

For each patient, 30° SD-OCT scans of the macula from three to four visits were analyzed carefully in the Retinal Imaging Research and Reading Center (RIRRC) at Wilmer, including scans at the first presentation of symptoms, a visit before treatment, and a post-treatment visit when available. The first visit was considered as the baseline and was labeled Day 0. A senior grader selected multiple cross sections that were representative of the retinal changes in each case. These selected sections were evaluated by two independent graders in the RIRRC who were informed of the patients' diagnosis but masked to the visits that were graded. Presence of hyperreflective signals, macular edema (ME), pigment epithelial detachment (PED), subretinal fluid (SRF), and any other abnormalities were assessed. The locations of hyperreflective signals were marked as being present in the following layers: nerve fiber layer (NFL), ganglion cell layer (GCL), inner plexiform layer (IPL), inner nuclear layer (INL), outer plexiform layer (OPL), outer nuclear layer (ONL), photoreceptor layer (PRL), RPE, and choroid. Any disagreement between the two graders was decided by a third senior grader. The TruTrack™ active eye tracking system in Spectralis SD-OCT allowed for comparative analysis of the same cross sections between the visits.

## Results and discussion

### Results

Five eyes of five patients were included. SD-OCT images from a total of 16 visits were reviewed, with a mean duration of 163 ± 61 days between baseline and last reviewed visit. Patient 5 had recurring disease, and only the latest recurrence of active disease was reviewed, since SD-OCT scans were not taken in the previous visits. The patient demographics and clinical findings are summarized in Tables 1 and 2, respectively.

**Table 1:** Demographics of study subjects

**Patient**	**Age**	**Gender**	**Study eye**	**Race**	**Histopathology**	**Time of observation (days)**
1	76	Male	OD	Caucasian	Large B-cell lymphoma	212
2	70	Male	OS	Caucasian	Large B-cell lymphoma	119
3	73	Male	OS	Caucasian	Large B-cell lymphoma	204
4	52	Female	OD	Caucasian	Large B-cell lymphoma	77
5	60	Female	OS	Caucasian	Large B-cell lymphoma	201

OD, right eye; OS, left eye; Time of observation, number of days between the baseline and last reviewed visit.

**Table 2:** Clinical findings over the observation period

**Patient**	**Slit lamp and fundus examination findings**
1	Anterior chamber cells and flare, vitreous cells and debris, macular edema
2	Vitreous cells and condensation, yellowish chorioretinal lesions
3	Keratic precipitates, anterior chamber cells and flare, vitreous cells, yellowish chorioretinal lesions
4	Vitreous cells, macular edema, yellowish chorioretinal lesions
5	Anterior chamber flare, vitreous haze, layered hypopyon

**Figure 1:**
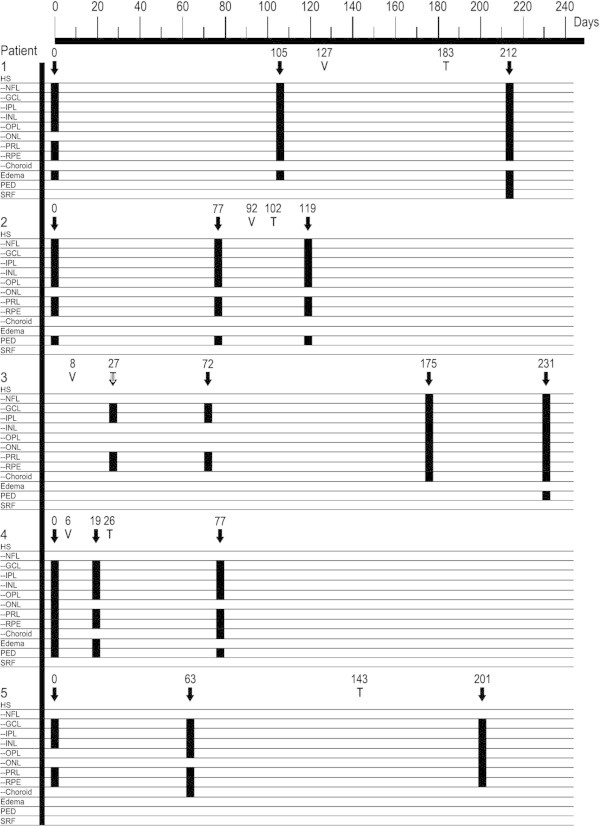
**Longitudinal SD-OCT finding.** Day 0 corresponds to the baseline visit for patients 1 to 4 and the first visit of the latest recurrence of intraocular lymphoma for patient 5. V indicates the date of the diagnostic vitrectomy. T indicates the initiation of therapy (systemic methotrexate and/or rituximab). SD-OCT images taken on the days marked with downward arrows were graded for ME, PED, SRF, and HS in retinal layers with positive finding being indicated by a black box.

**Figure 2:**
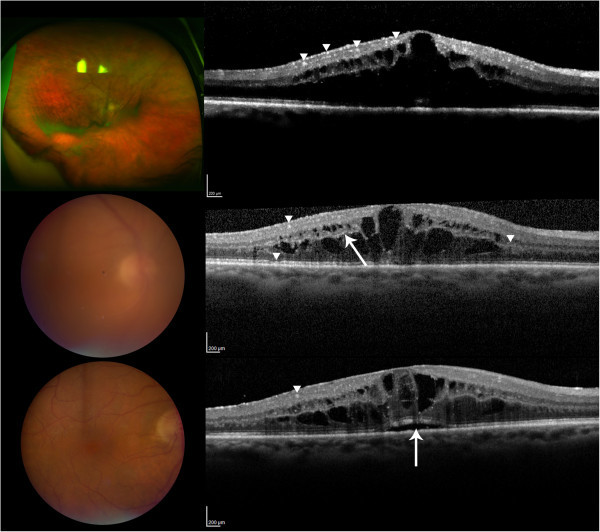
**Patient 1.** (Top) Day 0, there are multiple hyperreflective signals in the inner retina, indicated by the arrowheads. ME is present. The Optos® wide-angle fundus image shows sheets of cells. (Center) Day 105, examples of hyperreflective signals are pointed by the arrowheads. The arrowhead on the right points to thickening of the OPL. The arrow indicates hyperreflective signals in OPL. Slight disruption of the RPE is pointed by the bottom arrowhead. Color fundus shows significant vitritis. (Bottom) Day 212, the arrow points to elevation of RPE due to an accumulation of SRF. A nodular hyperreflective signal is pointed by the arrowhead. The view of the fundus is clear following vitrectomy.

**Figure 3:**
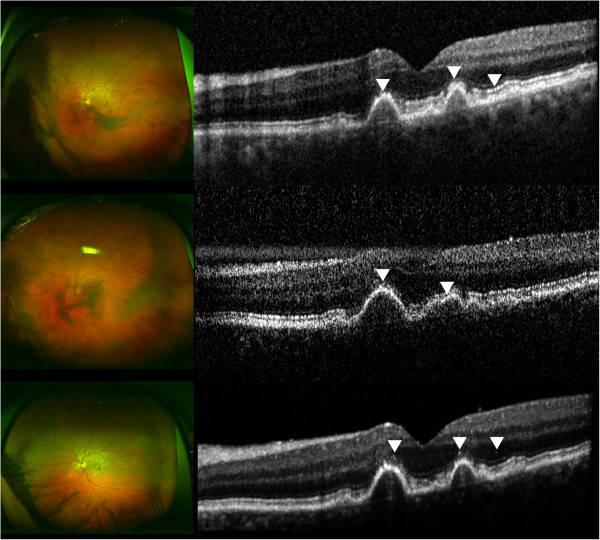
**Patient 2.** (Top) Day 0, multiple nodular hyperreflective signals in the outer retina are indicated by the arrowheads. The rightmost arrowhead points to hyperreflective signals at PRL and RPE level. RPE disruption is present due to drusen. There are sheets of vitreous cells shown in the Optos® wide-angle image. (Center) Day 77, arrowheads indicate hyperreflective changes at PRL and RPE. Severe vitritis as shown in the left fundus contributes to the noisy SD-OCT scan. Optos® wide-angle image shows increased vitreous inflammation. (Bottom) Day 119, arrowheads point to nodular hyperreflective signals that may be lymphomatous infiltrates. The view of the fundus is clearer post-vitrectomy.

**Figure 4:**
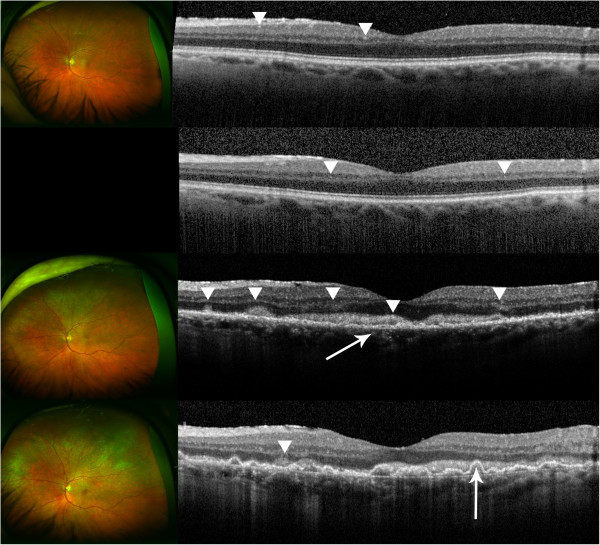
**Patient 3.** (Top) Day 27, the image appears normal with few abnormal hyperreflective signals that may indicate infiltrates as indicated by the arrowheads. Optos® wide-angle image shows clear vitreous following vitrectomy. (Center, upper) Day 72, the retina remains unchanged with a few hyperreflective signals as indicated by the arrowheads. No fundus image was available. (Center, lower) Day 175, evident hyperreflective signals are pointed by arrowheads. Layers are distorted, and RPE is disrupted as shown by the arrow. Inner segment/outer segment interface is no longer visible. There are new yellow lesions seen in the Optos® wide-angle image. A creamy lesion in the fovea is evident. (Bottom) Day 231, an increased amount of hyperreflective signals is found throughout the retina, and the overall reflectivity has also increased. RPE disruption has developed into pigment epithelial detachment as indicated by the arrow. Hyperreflective foci are found in the choroid as well. Optos® wide-angle image shows an increased degree of infiltration.

**Figure 5:**
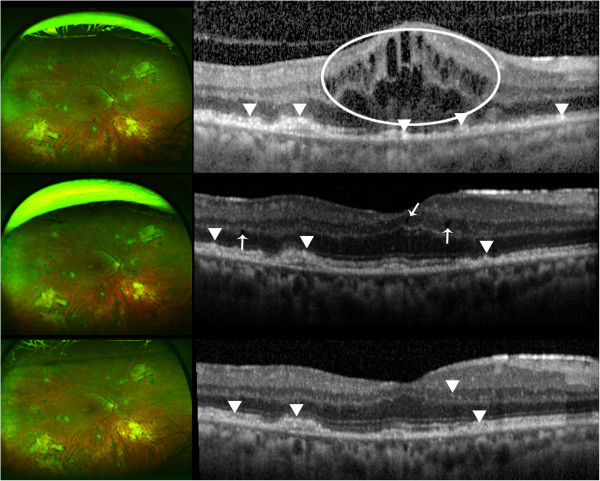
**Patient 4.** (Top) Day 0, macular edema with intracystic changes is circled. Arrowheads indicate hyperreflective signals at the PRL and RPE. The OCT scan was done before vitrectomy; there is significant noise in the scan. Optos® wide-angle image is from Day 14, following vitrectomy. (Center) Day 19, the arrows indicate intraretinal fluids. Lesions at the PRL and RPE are pointed by the arrowheads. Optos® wide-angle image from the same day shows stable retina. (Bottom) Day 77, hyperreflective signals at the RPE pointed by the arrowheads have arguably thickened. Hyperreflective signals are also seen in the inner retina. An arrowhead points to an example in the inner nuclear layer. No changes are seen in the Optos® wide-angle image from the same day.

**Figure 6:**
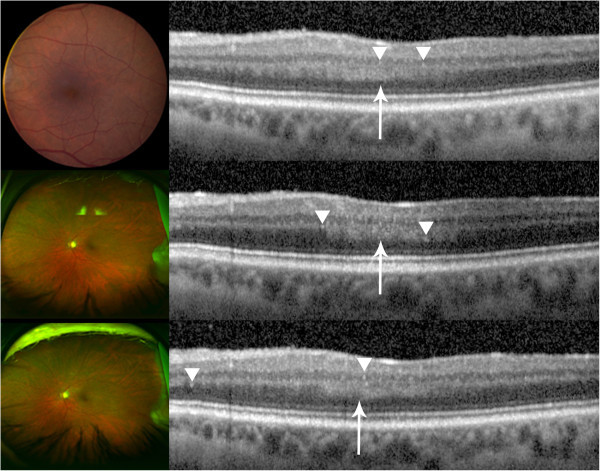
**Patient 5.** Unusually thick OPL is noted by an arrow and hyperreflective signals are indicated with arrowheads. (Top) Day 0, color fundus photograph is from a visit that took place a month before Day 0; no significant change in the fundus examination was noted in between the two visits. (Center) Day 63, an increase in hyperreflective signals is seen, which correlates to the increase in disease activity. (Bottom) Day 201, an increase in hyperreflective signals was noted, which may correlate to a significant increase in disease activity, manifested also by increased hypopyon, on this visit.

Figure 1 summarizes the SD-OCT findings for all patient visits included in this study. Hyperreflective signals appeared as nodules or bands, found across the retina, RPE, and/or choroid. Figures 2,3,4,5,6 correspond to the course of patients 1 to 5, respectively. Each figure shows the disease progression in one of the graded SD-OCT images, as well as color fundus or Optos® ultra-wide-field images from the corresponding visits.

#### Clinical description of PIOL manifestation

In the following descriptions and corresponding figures, Day 0 corresponds to the first visit with symptoms and signs of PIOL in the study eye, with the exception of patient 5. Patient 5 had recurring PIOL, and Day 0 corresponds to the first visit of the latest recurrence.

*Patient 1* initially presented with severe vitreous inflammation affecting the vision in the right eye, which subsided with anti-inflammatory drugs in the follow-up visits. ME was present throughout the visits. He had had large B-cell intraocular lymphoma in the left eye in the past, which had not been documented with SD-OCT. Repeated evaluations of the CNS during the manifestation in the right eye did not reveal active CNS lymphoma. The patient was treated with systemic MTX and RTX. On SD-OCT, hyperreflective signals were found in the inner and outer retina, as well as RPE. SRF developed with disease progression (Figure 2).

*Patient 2* had a history of PCNSL and presented with vitreous cells and a large vitreous condensation in the left eye. During the follow-up visit, sheets of vitreous cells became more pronounced, which is commonly observed in intraocular lymphoma [12]. The diagnosis of PIOL was confirmed by a biopsy of the vitreous obtained via a vitrectomy, and the patient was treated systemically with MTX and RTX. The patient also has bilateral age-related macular degeneration (AMD). PED from AMD, as well as hyperreflective signals in the retina and RPE were found on SD-OCT (Figure 3).

*Patient 3* presented with mutton fat keratic precipitates, anterior chamber cells and flare, and clumps of vitreous cells in the left eye. Clinical findings and the patient's history of PCNSL were indicative of PIOL, as confirmed by vitrectomy. Subretinal infiltrates were followed 5 months after baseline evaluation. In addition to systemic MTX and RTX, the patient also received one injection of intravitreal MTX due to disease progression. SD-OCT initially revealed few hyperreflective signals in nodular shape, consistent with infiltrates in the retina and RPE. With disease progression, RPE disruption and hyperreflective bands in the retina emerged (Figure 4). The case report of this patient by Liu et al. has been published [11].

*Patient 4* was referred to the Wilmer Eye Institute after being treated with topical steroids for persistent ME. The patient had a history of PCNSL. On examination, the patient had sheets of vitreous cells in the right eye. Following diagnostic vitrectomy which revealed the presence of lymphomatous cells, systemic MTX and RTX were given. SD-OCT revealed ME, PED, and hyperreflective signals, especially notable at the RPE. Number of layers with hyperreflective dots decreased following vitrectomy (Figure 5).

*Patient 5* had recurrent bilateral biopsy-proven PIOL and PCNSL for 5 years. Each recurrence had been effectively treated with systemic MTX and RTX. The recurrences were marked by keratic precipitates, hypopyon, vitreous haze, and/or ME. In the latest recurrence, the patient presented with mild anterior chamber flare and mild vitreous haze in the left eye. During the follow-up visits, layered hypopyon was present. The patient was treated with systemic MTX and RTX. SD-OCT findings included thickened OPL and hyperreflective signals in the inner and outer retina, as well as RPE (Figure 6).

## Discussion

Advancements in imaging technology in general and OCT in particular during the past decade have allowed for non-invasive identification of anatomical changes very much at the tissue level. In this study, we aimed to characterize the longitudinal changes in the retina with PIOL visualized by SD-OCT during the course of the disease. Previously reported OCT findings in PIOL include ME, hyperreflective materials at the RPE and outer retina, RPE disruption, and subretinal deposits which are similar to the abnormalities found in our study [8,11]. In particular, SD-OCT images of patient 4 were notably similar to those of the one case reported by Liang et al. with hyperreflective nodules at the RPE [10]. Our most common findings are abnormal hyperreflective signals that appeared as dots, nodules, and bands, with the highest frequency of appearance in GCL, IPL, PRL, and RPE as shown in Figure 1. Although hyperreflectivity in the inner retina was not discussed in previous OCT studies of PIOL, we identified hyperreflective signals in the inner retina of all patients with PIOL, which is most clearly illustrated in Figure 2. Chan et al. have shown that in murine models of PIOL, lymphoma cells inoculated in the vitreous can migrate and gather between RPE and retina, suggesting that lymphoma cells can be found across the retina [13].

Despite the consistent findings of hyperreflective signals on SD-OCT images of patients with PIOL that have been reported in the literature, the specific nature of the hyperreflective material seen within the inner and outer retina is still to be evaluated, and becomes a challenge without direct supporting histopathological evidence. Additionally, hyperreflective signals can be overestimated when inherent noise and vitreous haze are present, and careful distinction should be made during the grading and evaluating process. Moreover, hyperreflective foci have been reported in numerous diseases, such as AMD and diabetic macular edema, and should be excluded as sources of findings seen in PIOL patients [14-16]. In the case of patient 3, the changes in SD-OCT with the progression of PIOL evidently appeared as hyperreflective bands in INL, OPL, and ONL with corresponding chorioretinal changes seen in fundus examinations. Patient 5 had an increase in activity of disease noted in the anterior segment that corresponded to an increase in hyperreflective lesions noted in the retina (Figure 6). On the other hand, patient 2 has bilateral AMD; hyperreflective nodules at the RPE level may have indicated lymphoma infiltrates or equally may have been representative of hyperreflective lesions that have been reported in intermediate AMD (Figure 3) [14]. Therefore, hyperreflective changes in SD-OCT should be interpreted in the context of clinical findings.

## Conclusions

Submacular infiltration of lymphoma cells may be visualized by high-resolution SD-OCT but should be differentiated from noise on a suboptimal quality scan or hyperreflective signals due to other pathologies. Our findings indicate that hyperreflective signals increased with disease activity, suggesting that SD-OCT may be employed to monitor the progression of PIOL, which may influence decision making in the management of the disease.

## Abbreviations

PIOL: primary intraocular lymphoma; PCNSL: primary central nervous system lymphoma; SD-OCT: spectral domain optical coherence tomography; ME: macular edema; PED: pigment epithelial detachment; SRF: subretinal fluid; MTX: methotrexate; RTX: rituximab; AMD: age-related macular degeneration; NFL: nerve fiber layer; GCL: ganglion cell layer; IPL: inner plexiform layer; INL: inner nuclear layer; OPL: outer plexiform layer; ONL: outer nuclear layer; PRL: photoreceptor layer; RPE: retinal pigment epithelium.

## Competing interests

The authors declare that they have no competing interests.

## Authors’ contributions

HJ coordinated the study and drafted the manuscript. YJS, DVD, and QDN conceived of the study and contributed to its design. QDN provided the data used in the study. YJS, RS, and MGB graded the retinal scans and helped to draft the manuscript. HJ, YJS, RS, MGB, DVD, and QDN participated in the analysis and interpretation. DF, MH, and HL conducted literature search and critical revision of the article. All authors read and approved the final manuscript.

## Authors’ information

QDN is the recipient of the Physician Scientist Award from the Research to Prevent Blindness. DF is the recipient of the award from Coordenação de Aperfeiçoamento de Pessoal de Nível Superior. DVD is the recipient of the Heed Ophthalmic Foundation Clinician-Scientist Award. YJS is the Director of the Ocular Imaging Research and Reading Center at the Stanley M. Truhlsen Eye Institute. RS, MH, HL and MGB are postdoctoral research fellows at the Retinal Imaging Research and Reading Center.
